# *Bacillus subtilis* PE7-Mediated Alleviation of Phosphate Starvation and Growth Promotion of Netted Melon (*Cucumis melo* L. var. *reticulatus* Naud.)

**DOI:** 10.3390/microorganisms12122384

**Published:** 2024-11-21

**Authors:** Seong Eun Han, Kil Yong Kim, Chaw Ei Htwe Maung

**Affiliations:** 1 Department of Agricultural Chemistry, Environmentally-Friendly Agricultural Research Center, College of Agriculture and Life Sciences, Chonnam National University, Gwangju 61186, Republic of Korea; tjddms1160@naver.com; 2 Department of Agricultural and Biological Chemistry, Environmentally-Friendly Agricultural Research Center, College of Agriculture and Life Sciences, Chonnam National University, Gwangju 61186, Republic of Korea; 3 Environmentally-Friendly Agricultural Research Center, College of Agriculture and Life Sciences, Chonnam National University, Gwangju 61186, Republic of Korea

**Keywords:** *Bacillus subtilis*, organic acids, phytase, P solubilization, mineralization, biofilm

## Abstract

Members of *Bacillus* species are able to enhance the level of available phosphorus (P) for plant absorption through mechanisms of P solubilization and mineralization. In our study, *B. subtilis* PE7 showed P-solubilizing activity in simple phosphate broth (SPB) medium, and acetic acid, iso-butyric acid, and iso-valeric acid were major organic acids responsible for the increase in soluble P and decrease in pH of SPB medium. In addition, strain PE7 released phytase on phytase-screening agar (PSA) medium, and analysis of semi-quantitative reverse transcription and polymerase chain reaction (sqRT-PCR) revealed that the *phyC* gene expression was the highest at 1 day after incubation. A low concentration of KH_2_PO_4_ in SPB medium induced more biofilm formation than a high concentration of KH_2_PO_4_. Strain PE7 showed swimming and swarming motilities in TY and TrA agar media. Under P starvation, inoculation with higher cell numbers of strain PE7 enhanced biomass and nutrient acquisition by melon plants, resulting in higher values of growth parameters and nutrient contents. Moreover, the persistence of bacterial cells on the root surface and in the rhizosphere of melon plants indicated colonization of the plants by strain PE7. Due to its capacity for P solubilization and mineralization, *B. subtilis* PE7 could be utilized as an alternative to synthetic fertilizer for P deficient-stress management in crop plantation.

## 1. Introduction

Phosphorus (P) is one of the essential nutrients for plants. It plays a key role in the regulation of lipid metabolism and physiological responses of plants, besides being an integral component of ATP, nucleic acids, and cell membranes, all of which are required exclusively for plant developmental processes [1,2]. Although a sufficient concentration of P, 30–50% in the organic form and 35–70% in the inorganic form of total P, exists in soil [3], plants take up less than 30% because much of the P in its soluble form, originating from fertilization, is converted in the soil into insoluble complexes owing to the high reactivity of phosphate anions with cations, particularly Ca^2+^ in alkaline soil and Fe^3+^ and Al^3+^ in acidic soil [4,5]. To maximize P availability, P fertilizers have been utilized for decades to boost efficient crop production, but growing concerns about P limitation in global agricultural fields has led to excessive use of P fertilizers. These continued inputs of fertilizer and manure P in excess of crop requirements have led to a build-up of soil P levels, which are of environmental concern [6]. To cope with the immobility of P in soil and reduce the application rates of P fertilizers, the utilization of plant-growth-promoting bacteria (PGPB) possessing P-solubilizing capacity has been considered as an eco-friendly strategy. Most PGPB possess dual characteristics, such as inorganic P solubilization and mineralization of organic P sources [7,8]. As a primary route of P mobilization, they release a variety of organic acids, whose hydroxyl and carboxyl groups chelate the cations (Ca^2+^, Fe^3+^, and Al^3+^) bound to the insoluble P compounds (hydroxyapatite or metal–P complex) through ligand exchange reactions, thereby providing ortho-phosphate (ortho-P) for plant absorption and utilization [3,8,9]. Furthermore, PGPB hydrolyze and mineralize organic forms of P (phytate and phosphate esters) by enzymatic degradation through the production of extracellular enzymes, such as alkaline phosphatase, acid phosphatase, and phytase [7,8]. Phosphatases elevate the ortho-P concentration for plant nutrition via the cleavage of P from the ester linkage of organic P substrates in the soil. However, the synthesis of phosphatases by these bacteria relies remarkably on the level of free phosphate as well as inorganic nitrogen [7,9]. In addition, phytase hydrolyzes the phosphate ester bond of the soil phytate (myo-inositol hexakisphosphate) and releases soluble P moieties, thereby accelerating P availability for plant uptake [10,11]. Diverse PGPB genera, including *Agrobacterium*, *Azotobacter*, *Arthrobacter*, *Burkholderia*, *Bacillus*, *Enterobacter*, *Pseudomonas*, *Serratia*, and *Thiobacillus*, have been recognized for their assistance in liberating P from inorganic and organic P sources in soil for plant growth [12,13]. Among several species of PGPB, *Bacillus* species are well documented for exerting impressive plant-growth-promoting (PGP) effects through an efficient supply of P and, thereby, elevating the nutrient availability of the host plant, leading to better improvement of plant growth [2,14]. These *Bacilli*, including *Bacillus subtilis*, *Bacillus licheniformis*, *Bacillus megaterium*, *Bacillus cereus* and *Bacillus velezensis* are common P solubilizers through the production of a variety of organic acids such as gluconic acid, citric acid, succinic acid, and acetic acid [15,16,17,18,19]. They enable the enzymatic mineralization of organic P sources in the soil to boost the efficiency of soluble P acquisition for plant uptake [20,21,22]. Under P-deficient conditions, many soil-originated *Bacillus* species assist P availability and manipulate plant growth via their surface-root-colonizing and endophytic growth patterns [12,23].

Melon (*Cucumis melo* L.) is known for its richness in sugar, vitamins, folic acid, citric acid, and potassium, as well as its sweetness and excellent fragrance, making it a high-value food for consumers [24]. Moreover, the nutritional contents in melon are beneficial for lowering hypertension and the risk of heart stroke and promoting eye and skin health [24,25]. In our previous study, we isolated a PGPB strain, *B. subtilis* PE7, from cabbage kimchi, and its PGP effect through the production of indole-3-acetic acid (IAA) on netted melon was demonstrated [26]. However, the early growth stage and fruit development of melons are seriously affected by their low P contents under P-starvation conditions in soils [27,28]. During the early development stage of melon plant, the nutrient accumulation in the fruit pulp and other fruit qualities, including the color, sugar content, and firmness, can vary depending on the P availability [28]. Therefore, the present study was conducted to test the hypothesis that *B. subtilis* PE7 solubilizes insoluble P, thereby alleviating phosphate starvation and improving melon growth under P-deficient conditions. The objectives of our study were to determine the in vitro P-solubilizing and -mineralizing activity of *B. subtilis* PE7 through its production of organic acids and phytase, to evaluate its in vitro biofilm formation and, finally, to verify the P-solubilizing capacity of *B. subtilis* PE7 for the growth improvement of netted melon.

## 2. Materials and Methods

### 2.1. Microbial Strains, Culture Conditions, and Inoculum Preparation

*B. subtilis* PE7 (accession number: KACC 92549P), isolated from kimchi as previously described by Han et al. [26], was used in the present study. The bacterial strain was sub-cultured on tryptone soy agar (TSA) medium at 30 °C for 2 days and used for further experiments. Simple phosphate broth (SPB) medium (per liter: glucose 10 g, (NH_4_)_2_SO_4_ 0.6 g, KCl 0.4 g, and Ca_3_(PO_4_)_2_ 5 g) was used for the quantitative determination of the phosphate-solubilizing activity and biofilm formation of strain PE7 [29]. For determining the phytase activity, phytase-screening agar (PSA) medium (per liter: D-glucose 20 g, sodium phytate 4 g, CaCl_2_ 2 g, NH_4_NO_3_ 5 g, KCl 0.5 g, MgSO_4_ 7H_2_O 0.5 g, FeSO_4_·7H_2_O 0.01 g, MnSO_4_·H_2_O 0.01 g, and agar 20 g; pH 7.0) was used [30]. For the motility test, TY medium (per liter: tryptone 5 g, yeast extract 3 g, and CaCl_2_·6H_2_O 1.3 g) and TrA medium (per liter: tryptone 10 g and NaCl 5 g) were selected [31,32], and a 10-fold-diluted medium of each was used to determine the motility of strain PE7.

### 2.2. Quantitative Analysis of P-Solubilizing Activity of B. subtilis PE7 by UV Spectrophotometry and Detection of Organic Acids by High-Performance Liquid Chromatography (HPLC)

The P-solubilizing activity of *B. subtilis* PE7 in SPB medium was quantitatively determined following the method by Pande et al. [33]. To obtain the bacterial cell suspension, a single colony of strain PE7 was cultured in 300 mL of SPB medium at 30 °C and 130 rpm for 4 days. After centrifugation at 4 °C and 12,000 rpm for 15 min, the pellet was collected, suspended in sterile distilled water (SDW), and washed twice with SDW. Then, 500 µL of the bacterial cell suspension (1.68 ± 0.03 × 10^8^ CFU mL^−1^) was inoculated again in 500 mL of SPB medium and incubated at 30 °C and 130 rpm for 30 days. Three separate flasks were used for this experiment, and the broth cultures were sampled at 0, 3, 9, 12, 18, 24, and 30 days after incubation (DAI). The pH of the collected samples was measured using a pH meter (Orion™ Star A211, Seoul, Republic of Korea), and the broth cultures were centrifuged at 4 °C and 12,000 rpm for 15 min. After filtering with a 0.2 μm syringe filter, the resulting supernatant (100 µL) was mixed with Barton’s reagent (250 µL), and the volume was made up to 5 mL with double-distilled water (DDW). After keeping it at room temperature for 10 min, the absorbance was measured at 430 nm using a UV spectrophotometer (Shimadzu, Kyoto, Japan). The amount of soluble P was determined based on the standard curve of KH_2_PO_4_.

The supernatants from 6, 12, and 24 DAI were filtered through a 0.2 µm syringe filter, and 20 µL of each supernatant was injected into a high-performance liquid chromatography (HPLC) system (Prominence Shimadzu Co., Tokyo, Japan), which was equipped with two Shim-pack SCR-102H columns (300 × 8.0 mm). The detection of organic acids was carried out in comparison with a standard organic acid kit (Sigma-Aldrich, St. Louis, MO, USA) under the following conditions: mobile phase; 4 mM *p*-toluene sulfonic acid; flow rate 0.8 mL min^−1^; temperature 40 °C.

### 2.3. Phytase Activity on Agar Medium

The phytase production of *B. subtilis* PE7 was determined on PSA medium containing different concentrations of phytate as the substrate [34]. The pre-inoculated broth culture (5 µL, 10^8^ CFU mL^−1^) of strain PE7 was dropped at the center of each PSA medium supplemented with different concentrations (500, 1000, 2000, and 4000 ppm) of phytate. Triplicate plates were incubated at 30 °C for 3 days, and the diameter of the clear zone around the bacterial colony was measured.

### 2.4. Phytase Gene Expression Analysis

*B. subtilis* PE7 was grown on PSA medium containing 4000 ppm of phytate at 30 °C for 3 days. RNA extraction was conducted with *B. subtilis* PE7 samples (1, 2, and 3 DAI) by using a total RNA extraction kit (Cat. No. IVT3001) (Invirustech, Gwangju, Republic of Korea). cDNA was synthesized with the extracted RNAs by using AccuPower RT PreMix (BioNEER, Daejeon, Republic of Korea). The cDNA synthesis conditions were set at 72 °C for 5 min, 8 °C for 20 s, 42 °C for 1 h, and 94 °C for 5 min. Polymerase chain reaction (PCR) amplification and gel electrophoresis were performed to determine the presence and size of the PCR product using *phyC* gene primers (phyC, Fw: 5′-ACAGGGAAAAAGGTGCGTGA-3′ and phyC, Rv: 5′-CATTTCGCCGAGATGCTGTG-3′) designed by the Primer 3 Plus software (http://www.primer3plus.com/index.html) (accessed on 5 November 2024). For the PCR reaction, a mixture containing 1 µL of each primer, 1 µL of cDNA (1:20 dilutions), 17 µL of ddH_2_O, and AccuPower PCR PreMix (BioNEER, Daejeon, Republic of Korea) was used. The PCR amplification conditions included an initial denaturation at 95 °C for 5 min, followed by 27 cycles of denaturation, annealing, and extension at 95 °C for 20 s, 55 °C for 30 s, and 72 °C for 60 s, and the final extension was performed at 72 °C for 5 min. To compare the expression patterns of the *phyC* gene, a semi-quantitative reverse transcription PCR (sqRT-PCR) method was used. The ribosomal gene, *rpsj* (201 bp), was used as the internal control. NC is the no-template negative control, whereas *B. subtilis* PE7 cells were used as the positive control (PC).

### 2.5. Effect of Different Concentrations of KH_2_PO_4_ on Biofilm Formation

Different concentrations of KH_2_PO_4_ in SPB medium were used to determine the intensity of biofilm formation of *B. subtilis* PE7 following the protocol by Ghosh et al. [35]. Cells of strain PE7 were harvested from the pre-inoculated broth culture by centrifugation (4 °C, 12,000 rpm, 15 min). The resulting cells were washed twice with SDW and re-suspended in SDW. The sterile SPB medium (5 mL) supplemented with different concentrations (0, 12.5, 25, and 50 µM) of KH_2_PO_4_ was added into the wells of a 6-well polystyrene plate. The bacterial cell suspension (50 µL, 10^8^ CFU mL^−1^) was inoculated into each well, and triplicate plates were incubated at 30 °C for 10 days. The broth cultures were carefully discarded with a pipette, and 5 mL of methanol was added to fix the biofilm in each well. After air-drying, the fixed biofilm was stained with 5 mL of crystal violet solution and incubated at room temperature for 15 min. The excess stain was removed by washing with SDW, and the violet stain was dissolved with 5 mL of 70% ethanol for 15 min. After filtering the eluted stain with a 0.2 μm syringe filter, the absorbance was measured at 595 nm using a UV spectrophotometer.

### 2.6. Motility Assay

Swarming and swimming motilities of *B. subtilis* PE7 were examined on two different media (TY and TrA agar media) by using 6-well cell culture plates following the method described by Lucero et al. [31]. Each medium containing different concentrations of agar (0.3% for swimming activity and 0.5% for swarming activity) was prepared. Cells of strain PE7 were collected as described in the above experiment, and the bacterial cell suspension (5 µL, 10^8^ CFU mL^−1^) was inoculated at the center of each medium. Triplicate plates were incubated at 30 °C for 48 h, and the diameter of the migrating colony of strain PE7 was measured at 0, 6, 12, 24, and 48 h after incubation.

### 2.7. Preparation of Hoagland’s Solution, Viable Cells, and Broth Culture of B. subtilis PE7 for Pot Experiment

Hoagland’s solution (per liter: KH_2_PO_4_ 0.439 g, KNO_3_ 0.5055 g, Ca(NO_3_)_2_·4H_2_O 1.181 g, MgSO_4_ 0.9859 g, and micronutrient solution 1 mL) was prepared with slight modification as a nutrient solution for melon plants [36]. One liter of micronutrient solution consisted of FeCl_3_ 0.5 g, MnCl_2_·4H_2_O 1.82 g, ZnSO_4_·7H_2_O 0.08 g, CuSO_4_·5H_2_O 0.02 g, Na_2_MoO_4_·2H_2_O 0.12 g, and H_3_BO_3_ 2.86 g; pH 7.00.

The pre-inoculated culture (1 × 10^8^ CFU mL^−1^) of *B. subtilis* PE7 was inoculated in a flask containing KH_2_PO_4_-excluded Hoagland’s solution with supplementation of Ca_3_(PO_4_)_2_ 0.5 g L^−1^, glucose 2 g L^−1^, and yeast extract 0.2 g L^−1^. The flask was incubated at 30 °C for 7 days, and the broth culture was used for the pot experiment.

To prepare fresh inoculum of *B. subtilis* PE7, a single colony of strain PE7 was cultured in tryptone soy broth (TSB) medium at 30 °C and 130 rpm for 2 days. The bacterial cells were harvested by centrifugation at 4 °C and 12,000 rpm for 15 min, and after washing twice with SDW, the cells were re-suspended in SDW. The cell numbers in the resulting bacterial cell suspension were determined on TSA medium by serial dilution and the plate count method. The final cell concentrations were adjusted to 2.08 × 10^6^ CFU mL^−1^ and 2.00 × 10^8^ CFU mL^−1^ for the pot experiment.

### 2.8. Pot Experiment

Melon (*C. melo* L. var. *reticulatus* Naud.) seeds were sown in a plastic tray filled with organic bed soil and watered routinely for 2 weeks. Then, identical two-leaf-stage melon seedlings were selected and transplanted into each pot containing 800 g of sterile sand. The experiment comprised five treatments: (1) negative control (N-Con), (2) positive control (P-Con), (3) inoculation of viable bacterial cells (10^6^ CFU mL^−1^) as BC6, (4) inoculation of viable bacterial cells (10^8^ CFU mL^−1^) as BC8, and (5) inoculation of bacterial broth culture (BBC). The experiment was set up with three replications, and five melon plants were used for each replication. At 6 days after transplantation, the plants were drenched with 50 mL (as first- and second-time treatments) and 100 mL (as third- and fourth-time treatments) of KH_2_PO_4_-excluded Hoagland’s solution for four consecutive weeks to fertilize the melon plants in the N-Con, BC6, and BC8 treatments. In these three treatments, each amount of Ca_3_(PO_4_)_2_ powder (0.025 g for first- and second-time treatments and 0.05 g for third- and fourth-time treatments) was scattered on the sand of each plant. For the BC6 and BC8 treatments, each 10 mL of the bacterial suspensions containing 2.08 × 10^6^ CFU mL^−1^ and 2.00 × 10^8^ CFU mL^−1^ of cells of strain PE7 were administered to the melon plants at every application time. In the P-Con treatment, each melon plant was drenched with the same amount of Hoagland’s solution, including KH_2_PO_4_. For the BCC treatment, the same volume of the 7-day-old broth culture of strain PE7 was applied to the melon plants at each application time. At 1 week after the final treatment, the 7-week-old melon plants were carefully uprooted, and the growth parameters of the melon plants (leaf number, length, and fresh and dry weights of shoots and roots) were determined. Dry weights of the shoots and roots of the melon plants were recorded after drying in an oven at 70 °C for 5 days.

### 2.9. Analysis of Nutrient Contents in Melon Plants

The essential nutrients in the melon plants from the above pot experiment were determined following the method by Han et al. [26]. Prior to analysis, the oven-dried melons of each treatment were finely ground using a mixer grinder. The dry powder (0.5 g) was added into a Kjeldahl flask along with 1 mL of H_2_O_2_ and 9 mL of HNO_3_ and kept overnight. The digestion of the sample proceeded in a heating block at 100 °C for 30 min, 150 °C for 30 min, 180 °C for 30 min, and 200 °C for 1 h. After cooling down, the total volume of the digested solution was completed to 100 mL with DDW. The sample was filtered through two layers of Whatman No. 6 filter paper, and the analysis of phosphorus (P), potassium (K), calcium (Ca), magnesium (Mg), sulfur (S), boron (B), copper (Cu), iron (Fe), manganese (Mn), boron (B), and molybdenum (Mo) was conducted using an inductively coupled plasma optical emission spectrometer (ICP-OES; Nexion 2000, PerkinElmer, Waltham, MA, USA). To estimate the total nitrogen (N) in the melon plants, the melon powder (50 mg) was added into a titanium capsule and analyzed using a vario MAX cube elemental analyzer (ELEMENTAR, Langenselbold, Germany).

### 2.10. Investigation of Root Colonization of B. subtilis PE7 Using Scanning Electron Microscopy (SEM)

The roots of 9-week-old melon plants of each treatment (P-Con, N-Con, BC6, BC8, and BCC) were sampled and carefully rinsed by soaking in tap water with gentle shaking. The roots were cut into 5 mm segments and pre-fixed at 4 °C overnight in glutaraldehyde solution (2.5%, *v*/*v*) prepared in 0.1 M phosphate buffer (pH 7.2). After washing thrice with the same buffer, the roots were post-fixed with 1% OsO_4_ at room temperature for 1 h and washed twice with sterile double-distilled water (SDDW). A series of ethanol solutions (35%, 50%, and 75% once, 95% twice, and 100% ethanol thrice) was used for the gradual dehydration of melon roots, and the final dehydration was carried out by a critical point dryer. The dried roots were coated with gold particles at 60 °C for 15 min by a sputter coater, and the root colonization was visualized using a field-emission scanning electron microscope (FESEM; Gemini 500, Zeiss, Oberkochen, Germany) and energy-dispersive spectrometer (EDS; X-MaxN 80; Oxford Instruments, Cambridge, UK).

### 2.11. Evaluation of B. subtilis PE7 Population in Pot

The population of *B. subtilis* PE7 in the rhizosphere of the melon plants was determined by serial dilution and the plate count method. Three melon plants from each treatment of strain PE7 (the BC6, BC8, and BBC treatments) were uprooted, and the sand adjacent to the roots of the melon plants was sampled and air-dried at room temperature. One gram of each sand sample was suspended in 9 mL of SDW and kept in an orbital shaker at 130 rpm for 30 min. Serial dilution was carried out, and the diluted sample was spread on TSA medium. After incubation at 30 °C for 2 days, colonies similar to strain PE7 were counted and recorded as colony-forming units (CFU) per gram of sand.

### 2.12. Statistical Analysis

Experimental data were subjected to analysis of variance (ANOVA) using the Statistical Analysis System (SAS) software (version 9.4; Raleigh, NC, USA). The mean comparison between the values of the data from the in vitro and pot experiments was examined at the *p* < 0.05 level by the least significant difference (LSD) test.

## 3. Results

### 3.1. P-Solubilizing Activity and Organic Acid Production of B. subtilis PE7

**Figure 1:**
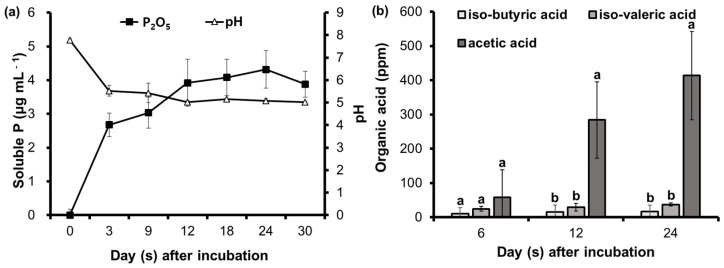
P-solubilizing capacity of *B. subtilis* PE7 in simple phosphate broth (SPB) medium showing the soluble P concentration and pH value during 30-day incubation period (**a**) and concentration of organic acids released by strain PE7 in SPB medium (**b**). The data in both graphs represent mean ± standard deviation of three replicates. Different letters on the bar graph indicate significant difference between organic acids at each incubation day as compared by the least significant difference (LSD) test at *p* < 0.05 level.

The P-solubilizing capacity of *B. subtilis* PE7 in SPB medium in terms of the amount of soluble P is shown in Figure 1a for an observation period of 30 days. The concentration of soluble P sharply increased to 2.67 ± 0.35 µg mL^−1^ at 3 DAI, with a decrease in the pH value to 5.53. Then, a steady increase in the soluble P concentration was observed from 9 DAI, reaching a maximum value of 4.32 ± 0.56 µg mL^−1^ at 24 DAI, and then the P amount decreased again at the end of the observation period. However, there was no remarkable change in the pH value from 12 DAI to the final observation day. The HPLC analysis of the cell-free supernatant of *B. subtilis* PE7 revealed the presence of three different types of organic acids, including iso-butyric acid, iso-valeric acid, and acetic acid (Figure 1b). The concentrations of organic acids during incubation showed a time-dependent increase. The highest concentrations of 413.42 ppm of acetic acid, 37.41 ppm of iso-valeric acid, and 16.71 ppm of iso-butyric acid were recorded at 24 DAI.

### 3.2. Phytase Activity of B. subtilis PE7 on PSA Medium and Phytase Gene Expression

**Figure 2:**
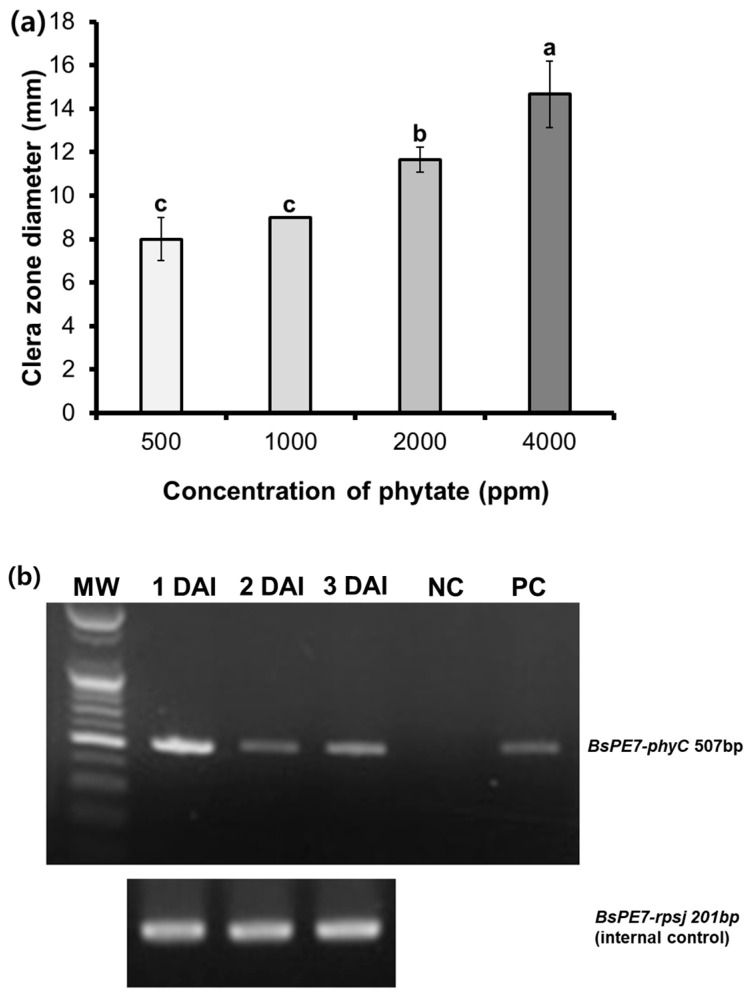
Phytase activity of *B. subtilis* PE7 depending on different concentrations of supplemented phytate in phytase-screening agar (PSA) medium (**a**). Expression patterns of *B. subtilis* PE7 *phyC* mRNA using the semi-quantitative RT-PCR method (**b**). cDNAs were synthesized with RNA samples from *B. subtilis* PE7 (1, 2, and 3 DAI) grown on PSA medium containing 4000 ppm of phytate. The ribosomal gene, *rpsj* (201bp), was used as the internal control. NC is the no-template negative control, whereas *B. subtilis* PE7 cells were used as positive control (PC). The data in the bar graph represent mean ± standard deviation of three replicates. Different letters on the bar graph indicate significant difference between treatments as compared by the least significant difference (LSD) test at *p* < 0.05 level.

The phytase activity of strain PE7 was dependent on the concentration of phytate supplemented into the agar medium (Figure 2a). Based on the length of the clear zone diameter recorded around the colonies in the growth medium supplemented with phytate, the release of phytase by strain PE7 increased with the increasing phytate concentration; the largest and smallest clear zone diameters (14.67 and 8.00 mm) were recorded in the growth medium added with 4000 and 500 ppm of phytate, respectively. Furthermore, the gel electrophoresis image (Figure 2b) indicated the appearance of the PCR product (approximately 507 bp), revealing the phytase gene expression of strain PE7. The level of the phytase gene (507 bp) was highly expressed at 1 DAI, although it decreased at 2 DAI and 3 DAI.

### 3.3. Effect of Soluble P Concentration on Biofilm Formation of B. subtilis PE7

**Figure 3:**
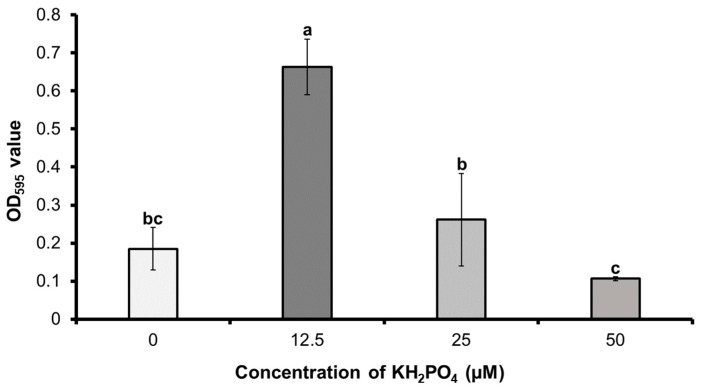
Biofilm formation of *B. subtilis* PE7 (as OD_595_ values) in simple phosphate broth (SPB) medium with different concentrations of KH_2_PO_4_. The data represent mean ± standard deviation of three replicates. Different letters on the graph indicate significant difference between treatments as compared by the least significant difference (LSD) test at *p* < 0.05 level.

As shown in Figure 3, the biofilm development of *B. subtilis* PE7 showed a negative correlation with the concentration of soluble P (KH_2_PO_4_) in the SPB medium. The biofilm formation of strain PE7 significantly decreased in the presence of a high concentration of soluble P. Strain PE7 induced the highest degree of biofilm formation in terms of the OD_595_ value (0.664) at the lower concentration of KH_2_PO_4_ (12.5 µM), whereas higher concentrations of KH_2_PO_4_ (25 and 50 µM) significantly reduced the biofilm formation, showing OD_595_ values of 0.262 and 0.106. Interestingly, the absence of KH_2_PO_4_ induced more biofilm formation than the treatments with 50 µM of KH_2_PO_4_, revealing a relatively higher OD_595_ value of 0.185.

### 3.4. Motility of B. subtilis PE7

**Figure 4:**
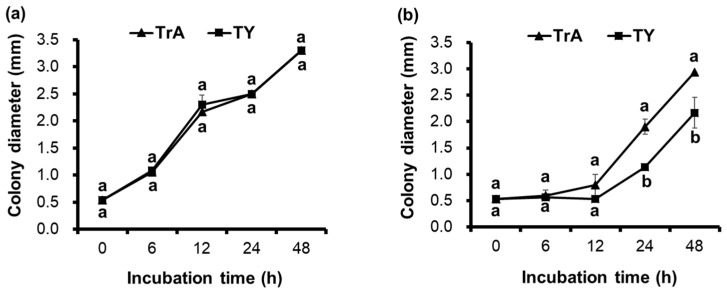

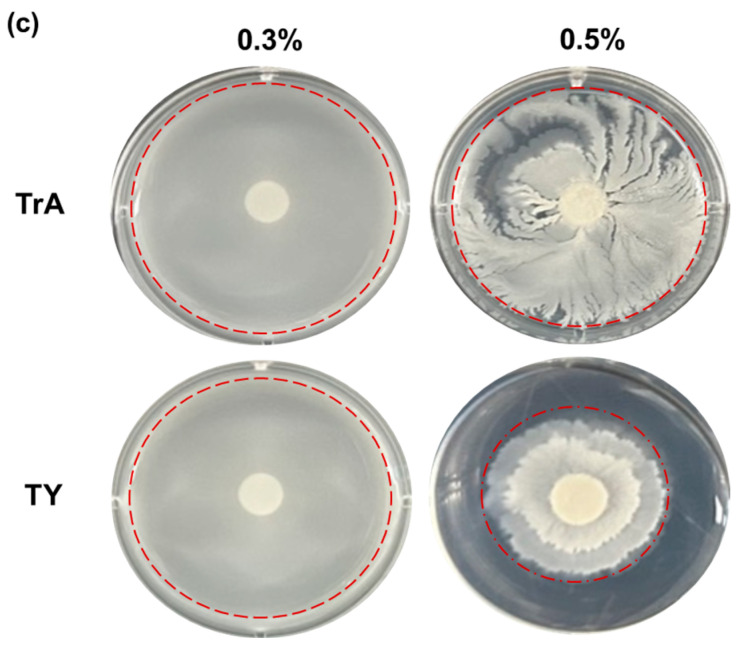
Time-course analysis of swimming (**a**) and swarming (**b**) abilities of *B. subtilis* PE7 and motility morphology of strain PE7 (**c**), including swimming (0.3% agar) and swarming (0.5% agar) on TrA and TY media. The data represent mean ± standard deviation of three replicates. Different letters on line graphs indicate significant difference in biofilm formation between two different media at each incubation time as compared by *t*-test at *p* < 0.05 level.

Figure 4a–c clearly show the swimming and swarming abilities of *B. subtilis* PE7 on both TrA and TY agar media. No significant difference in the swimming ability of strain PE7 was observed between the two media. However, strain PE7 showed statistically more proficient swarming motility on TrA agar medium than on TY agar medium during the late incubation period.

### 3.5. Effect of Cell Concentration and Broth Culture of B. subtilis PE7 on Melon Growth

Table 1 clearly shows that *B. subtilis* PE7 positively affected melon growth through its P-solubilizing activity, resulting in higher growth parameters of the melon plants when compared to the plants of the N-Con treatment. Due to sufficient soluble P, the plants in the P-Con treatment exhibited the highest values for all growth parameters except root dry weight. However, in the N-Con treatment, the plants were severely affected by the absence of a soluble P source, resulting in stunted growth with the lowest values of the growth parameters. Although there was no statistical difference in the root fresh weight between the P-Con and BC8 treatments, the inoculation of viable cells of *B. subtilis* PE7 (10^8^ CFU mL^−1^) remarkably induced the root biomass, leading to the highest root dry weight (0.72 ± 0.20 g), which was 1.36-fold higher than that of the P-Con treatment. Moreover, the values of the growth parameters in the BC8 treatment were significantly greater than those in the N-Con treatment. The treatment with a lower cell concentration of *B. subtilis* PE7 (the BC6 treatment) did not show a significant growth-promoting effect on the melon plants as compared to the N-Con treatment. Inoculation of *B. subtilis* PE7 broth culture (the BBC treatment) resulted in higher values of the length and dry weight of shoots than the N-Con treatment, despite showing similar values of other growth parameters in these two treatments. There was no remarkable difference in root lengths among all treatments.

### 3.6. Macronutrient and Micronutrient Contents in Melon Plants

The treatment with higher viable cell numbers of *B. subtilis* PE7 (the BC8 treatment) contributed remarkably to the uptake efficiency of macro- and micronutrients by the melon plants as compared to the other bacterial treatments (Table 2 and Table 3). The P-Con treatment resulted in the highest values of the nutrient content in the melon plants among the treatments. However, the insufficiency of a soluble P source intensely disturbed the nutrient uptake by the melon plants in the N-Con treatment, resulting in lower values of the nutrient contents except N and S contents as compared to the P-Con treatment. The inoculation of viable cells of strain PE7 adequately facilitated nutrient absorption by the melon plants in the BC8 and BBC treatments, as in both of these treatments, the values of the macro- and micronutrients were higher than those of the N-Con treatment, except for the values of the N, S, and B contents. There was no remarkable difference in the macronutrient content between the BC6 and N-Con treatments, except for the S content.

### 3.7. Root Colonization Pattern of B. subtilis PE7

**Figure 5:**
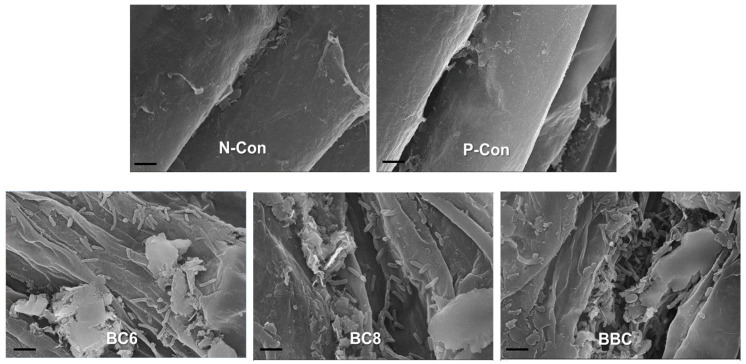
Scanning electron micrographs of melon root surfaces colonized by vegetative cells of *B. subtilis* PE7 in BC6, BC8, and BBC treatments as compared to the root surface in N-Con and P-Con treatments, indicating the absence of bacterial cells. The scale bar represents 2 µm. (P-Con: positive control, N-Con: negative control, BC6: treatment with viable cells (10^6^ CFU mL^−1^) of *B. subtilis* PE7, BC8: treatment with viable cells (10^8^ CFU mL^−1^) of *B. subtilis* PE7, BBC: treatment with broth culture of *B. subtilis* PE7).

The existence of vegetative cells of *B. subtilis* PE7 on the melon root surfaces of the bacterized treatments was revealed by SEM in our study (Figure 5). The micrographs of rod-shaped bacterial cells in the BC6 and BC8 treatments indicated an effective attachment and tight adherence to the root surfaces by the viable cells of *B. subtilis* PE7. The cells of strain PE7 abundantly colonized on the melon roots, forming clusters in the BBC treatment. However, no bacterial inhabitants were observed on the root surfaces of the melon plants in the N-Con and P-Con treatments.

### 3.8. Population Density of B. subtilis PE7 in the Pot Soil of Melon Plants

**Figure 6:**
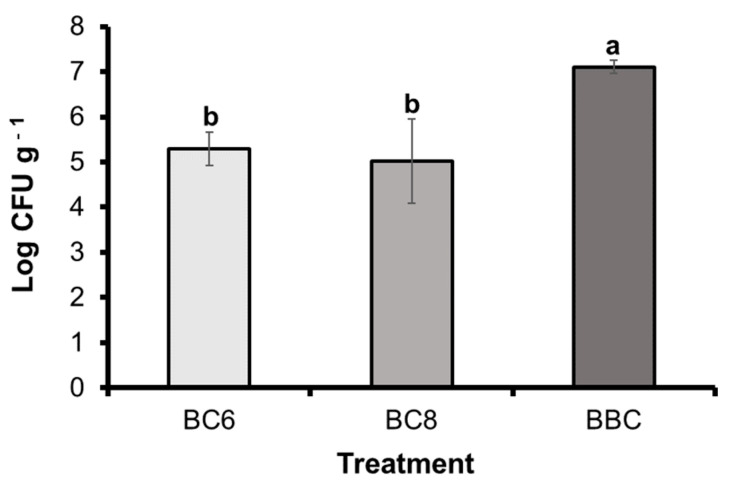
Population of *B. subtilis* PE7 in the rhizosphere sand of melon plants in BC6, BC8, and BBC treatments at 5 weeks after transplantation. The data represent mean ± standard deviation of three replicates. Different letters on the graph indicate significant difference between treatments as compared by the least significant difference (LSD) test at *p* < 0.05 level. (BC6: treatment with viable cells (10^6^ CFU mL^−1^) of *B. subtilis* PE7, BC8: treatment with viable cells (10^8^ CFU mL^−1^) of *B. subtilis* PE7, BBC: treatment with broth culture of *B. subtilis* PE7).

The results of the population of strain PE7 in the melon rhizosphere of the BC6, BC8, and BBC treatments are presented in Figure 6. The treatments with viable cells of strain PE7 showed a cell density of 10^5^ CFU g^−1^ (1.94 ± 0.71 × 10^5^ CFU g^−1^ in the sand of the BC6 treatment and 1.03 ± 0.15 × 10^5^ CFU g^−1^ in the BC8 treatment). However, the sand of the melon plants treated with the broth culture of strain PE7 exhibited a 100-fold higher cell density of strain PE7 (1.28 ± 0.69 × 10^7^ CFU g^−1^) than either of the BC6 or BC8 treatments.

## 4. Discussion

*Bacillus* species have been documented as a viable option for alleviating the stress induced by the depletion of P, as they possess both P-solubilizing and -mineralizing capabilities [22,37]. Therefore, the application of PGPB, including *Bacillus* species, has attracted much attention as an eco-friendly solution for the limitation of crop production due to P deficiency rather than using phosphate fertilizers [2,12]. *Bacillus* species are known to release diverse types of organic acids to solubilize complex forms of insoluble P in soil and elevate P availability for plant growth [18]. In the present study, *B. subtilis* PE7 solubilized insoluble P in SPB medium through the production of organic acids. Under in vitro conditions, three kinds of organic acids (iso-butyric acid, iso-valeric acid, and acetic acid) were mainly involved in the solubilization of insoluble P by strain PE7 in our study. Due to the secretion of these organic acids by strain PE7, the concentration of soluble P increased with a concomitant decrease in the pH of the growth medium.

The P-solubilizing capacity of PGPB and the amount of soluble P released can vary depending on the type and strength of organic acids [38]. In addition, the potency of P solubilization is highly correlated with the number of carboxylic groups of the released organic acids. Di- and tri-carboxylic acids, such as oxalic acid and citric acid, are better at chelating cations and converting bound phosphate into a soluble form than monocarboxylic acids like gluconic acid [39]. Therefore, the low concentration of soluble P in our study could be associated with the secretion of weak monocarboxylic acids by strain PE7. Moreover, Tahir et al. [15] demonstrated that the carbon source is also an important factor for the amount and type of organic acids produced by different P-solubilizing bacteria, including *Bacillus* strain T-34, and that various organic acids, including acetic acid, citric acid, and gluconic acid, are the main mediators of P solubilization by these strains. Conversely, *Bacillus* species are able to produce diverse hydrolases, such as phosphatases and phytases, which are responsible for the mineralization of organic P and phytate in soil [12]. Microbe-derived phytases are essential to catalyze and liberate inorganic phosphate from phytate in soil, contributing to the enhancement of a soluble P source for plant developmental processes [2,12]. Apart from the enhancement of the P level for plants, these microbe-derived phytases are utilized in numerous sectors, including soil remediation and the animal and human food industries, as well as the paper and pulp industries [40]. Different strains of *B. subtilis* were revealed as a natural reservoir of phytase to promote soil phytate P solubility for plant absorption, showing their strong potential to promote the growth of different plant species [2,22,41]. In addition, our study also demonstrated a concentration-dependent enhancing effect of supplemented phytate on the phytase activity of strain PE7 on the PSA medium. This result of the phytase activity indicates the possibility of strain PE7 for phytate mineralization in soil.

The expression patterns of *phyC* mRNA at different time points indicated that the highest expression level of *phyC* mRNA was observed at 1 DAI, followed by a decline on subsequent days (Figure 2b). These expression patterns suggest that the incubation period seemed to negatively regulate the *phyC* mRNA expression on the PSA medium due to the depletion of its substrate, phytate as the substrate in solid agar medium was fixed and not suspended. The phytase synthesis of strain PE7 in our study might have been modulated by the availability of the substrate under the substrate-limited conditions. Such *phyC* gene regulation highlights the adaptive responses of *B*. *subtilis* PE7 to fluctuating nutrient environments, optimizing its enzymatic output to enhance phytate mineralization during initial growth phases.

Under biotic and abiotic stress conditions, PGPB, including *Bacillus* species, structurally organize themselves into multicellular communities through adherence to each other by forming an extracellular matrix called biofilm [42,43,44]. Biofilm formation by PGPB is a prominent trait for successful persistence in rhizosphere soil and surface colonization on plant roots, and it also acts as a protective barrier for tolerance to toxic agents and extreme environmental conditions [42]. Ali et al. [45] revealed that the biofilm development of *Bacillus* species allows them to survive and release important active metabolites under salt stress. In our study, *B. subtilis* PE7 was able to generate biofilm formation in the growth medium with different KH_2_PO_4_ concentrations. However, an adequate amount of soluble P caused a reduction in the biofilm-forming capacity of strain PE7 in the present study. The highest concentration (50 µM) of KH_2_PO_4_ in the growth medium induced less biofilm formation than low or no KH_2_PO_4_. These results indicate that under the P-deficient conditions, strain PE7 was prompted to resist the nutrient-limited stress and formed a biofilm to survive in the growth medium. In similar studies, lower levels of soluble phosphate promoted a higher degree of biofilm development by *Burkholderia* species and *Bacillus* species [35,46]. Moreover, strains of *B. subtilis* were capable of migrating in an aqueous or semi-solid environment through their flagella-mediated swimming and swarming motilities [47,48]. These features play a vital role in helping bacteria reach more favorable sites for nutrients or plant-signaling molecules [49]. Our study demonstrated that *B. subtilis* PE7 showed its potential swimming and swarming activities on the different tested media. The collective cell movement of strain PE7 on the media containing 0.3% and 0.5% agar revealed its two patterns of motility. Gao et al. [48] evinced the swarming motility of *B. subtilis* SWR01 as a key mode of migration during the root colonization of tomato plants. Therefore, the swarming behavior of strain PE7 could enhance its initial movement, leading to its successful adherence and efficient surface colonization.

The plant-available forms of P (H_2_PO_4_^−^ and HPO_4_^2−^) are limited in agricultural soils [50]. As a result of the frequent application of P fertilizers, the highly soluble mono-calcium phosphate, Ca(H_2_PO_4_)_2_, in these P fertilizers rapidly fixes with the cations of other minerals, such as Fe and Al in acidic soil and Ca in alkaline soil, leading to the accumulation of insoluble P forms (FePO_4_, AlPO_4_, and Ca_3_(PO_4_)_2_) [50]. Due to the abundance of Ca in alkaline soil, the Ca(H_2_PO_4_)_2_ of P fertilizer reacts sequentially with calcium carbonate, (CaCO_3_), causing a gradual decrease in its solubility, and it then transforms into Ca_3_(PO_4_)_2_, the plant-unavailable form [50]. Fu et al. [51] demonstrated that salt–alkali stress significantly affects melon growth, resulting in a reduction in melon yield and quality. Therefore, Ca_3_(PO_4_)_2_ was used as the insoluble P substrate in our study to determine the P-solubilizing and plant-growth-promoting capacities of *B. subtilis* PE7 in melon plants. As P is an important macronutrient for plant growth and development, P-deficient conditions significantly affect the morphology and physiology of melon, contributing to stunted growth and reduced quality [28,52]. Moreover, the size and quality of melon fruit, including color, skin thickness, firmness, and sugar and phytonutrient contents, can vary depending on the P availability during the early stages of melon plant growth [28]. In the present study, the inoculation of higher numbers of viable cells or the broth culture of *B. subtilis* PE7 improved the melon growth in the BC8 and BBC treatments under P-deficient conditions. The melon plants in the BC8 and BBC treatments showed greater values of the growth parameters than those in the N-Con treatment. These results could be associated with the performance of strain PE7. By solubilizing Ca_3_(PO_4_)_2_ through the production of organic acids and thereby supplying soluble P for easy uptake of the melon plants, strain PE7 led to an improvement in the overall plant growth under P starvation. Our results are consistent with those of Dong et al. [53], who suggested that the inoculation of an increasing concentration of viable cells of *B. cereus* DW019 (10^9^ CFU mL^−1^) induced a significant growth improvement, including the yield and quality, of cherry tomato. However, lower cell numbers (10^6^ CFU mL^−1^) of strain PE7 could be insufficient in solubilizing insoluble P and supporting the required P for melon plants, as there was no statistical difference in the melon growth parameters between the N-Con and BC6 treatments. Inadequate cell numbers seemed to be a limiting factor in achieving a higher P solubilizing performance of strain PE7, which, in turn, reduced the root and shoot growth in the BC6 treatment as compared to the BC8 treatment.

In addition, significant root development in terms of the highest root dry weight in the BC8 treatment indicates that beyond P solubilization, strain PE7 seems to release other plant-growth-stimulating substances, including phytohormones such as IAA. Our previous study revealed IAA as a key player in melon growth, and the IAA content in the broth culture of strain PE7 significantly stimulated the well-developed root system and elevated nutrient absorption for the entire plant growth [26]. As a consequence of the growth enhancement of melon plants by strain PE7 through accelerating the P availability, the plants in the BC8 and BBC treatments displayed improved nutrient uptake as compared to the N-Con and BC6 treatments, attaining higher values of most of the macro- and micronutrients. However, due to the sufficient soluble P source, the plants in the P-Con treatment showed the best overall growth and nutrient content in our study. Likewise, in a study by Jensen et al. [22], the inoculation of *B. subtilis* ALC_02 in P-limited soil triggered an improved P acquisition, resulting in an increase in the size and P content of tomato plants.

Root colonization is an important mechanism by which PGPB demonstrate their PGP effects through their successful establishment for plant growth [54,55]. Motility and biofilm formation are important bacterial features for their successful attachment and persistence on plant roots, which, in turn, influence their PGP abilities and biocontrol efficacy [54]. The scanning electron micrographs revealed that *B. subtilis* PE7 tightly colonized on the melon roots of the bacterized treatments (BC6, BC8, and BBC treatments) in our study. The rod-shaped cells of strain PE7 consistently distributed and adhered to the root surface of the melon plants in these treatments, indicating their firm establishment. Our in vitro assays evinced that strain PE7 possesses swarming motility in two different media, as well as biofilm formation in the presence or absence of soluble P. Therefore, under P-deficient conditions, strong colonization of strain PE7 could be responsible for reducing P-limited stress, as well as providing the required soluble P for melon growth. Moreover, a large population of strain PE7 was observed in the pot sand of the melon plants in all the bacterized treatments. In the natural environment, the high competence of PGPB in the rhizosphere region plays a significant role in achieving consistent and desired PGP effects [55]. A large number of inoculated PGPB could lead to the desired effects when in competition with soil microbes [56]. The presence of abundant numbers of strain PE7 in the rhizosphere, as well as on the root surface of melon plants, could be one of the reasons for the growth enhancement of the melon plants in our study. Therefore, the higher growth parameters and greater nutritional values of the melon plants in the BC8 and BBC treatments revealed the capability of strain PE7 in regulating P-starvation stress and directly improving plant nutrition. Other PGP properties of strain PE7, such as successful root colonization, could also be involved as another mode of action for melon growth enhancement by strain PE7 under P-deficient conditions.

## 5. Conclusions

The important PGP traits of *B. subtilis* PE7, including P solubilization and mineralization, biofilm development, and root and rhizosphere colonization, were revealed in our study. Our pot experiment also demonstrated the performance of *B. subtilis* PE7 by manipulating P stress in melon plants through their direct supply of available P, which, in turn, improved the nutrient contents in the melon plants. These results confirmed the hypothesis of our study, showing that strain PE7 was an effective P solubilizer for the mitigation of P stress and growth enhancement of melon plants under P-limited conditions. Therefore, *B. subtilis* PE7 could be utilized as a potential inoculant for the development of commercial biofertilizers in the management of P deficiency and crop yield improvement. In future studies, the tolerance of *B. subtilis* PE7 to other abiotic stresses, such as drought and salinity, should be evaluated under greenhouse and field conditions. Their abiotic stress response and specific modes of action should also be assessed by advanced molecular techniques.

## Figures and Tables

**Table 1:** Effect of viable cells and broth culture of *B. subtilis* PE7 on melon growth at 5 weeks after transplantation.

Treatment *	Length (cm)		Fresh Weight (g)		Dry Weight (g)		Leaf Number
	Shoot	Root	Shoot	Root	Shoot	Root	
P-Con	34.68 ± 1.43 ^a^	32.30 ± 5.77 ^a^	30.27 ± 1.47 ^a^	7.68 ± 0.93 ^a^	3.51 ± 0.13 ^a^	0.53 ± 0.08 ^b^	11.00 ± 0.00 ^a^
N-Con	25.27 ± 1.57 ^d^	28.37 ± 1.57 ^a^	18.67 ± 1.16 ^c^	5.82 ± 0.69 ^b^	2.53 ± 0.22 ^cd^	0.43 ± 0.04 ^b^	8.83 ± 0.41 ^cd^
BC6	27.18 ± 0.41 ^c^	31.92 ± 5.56 ^a^	19.59 ± 1.91 ^c^	6.11 ± 0.79 ^b^	2.68 ± 0.28 ^cd^	0.45 ± 0.07 ^b^	9.17 ± 0.41 ^bcd^
BC8	30.08 ± 1.41 ^b^	28.70 ± 4.08 ^a^	23.68 ± 2.16 ^b^	7.60 ± 0.87 ^a^	3.16 ± 0.34 ^b^	0.72 ± 0.20 ^a^	9.67 ± 0.52 ^b^
BBC	29.15 ± 1.78 ^b^	31.75 ± 3.35 ^a^	19.67 ± 0.87 ^c^	6.48 ± 0.68 ^b^	3.01 ± 0.22 ^b^	0.49 ± 0.07 ^b^	9.33 ± 0.52 ^bc^

* P-Con: positive control, N-Con: negative control, BC6: treatment with viable cells (10^6^ CFU mL^−1^) of *B. subtilis* PE7, BC8: treatment with viable cells (10^8^ CFU mL^−1^) of *B. subtilis* PE7, BBC: treatment with broth culture of *B. subtilis* PE7. Data represent mean ± standard deviation of three replicates. Different letters in the same column indicate significant difference between treatments as compared by the least significant difference (LSD) test at *p* < 0.05 level.

**Table 2:** Effect of viable cells and broth culture of *B. subtilis* PE7 on macronutrient uptake in melon plants at 5 weeks after transplantation.

Treatment *	Macronutrient (mg plant^−1^)					
	N	P	K	Mg	Ca	S
P-Con	69.26 ± 5.40 ^a^	1.83 ± 0.18 ^a^	129.73 ± 1.69 ^a^	22.35 ± 0.46 ^a^	106.82 ± 1.25 ^a^	23.09 ± 11.56 ^a^
N-Con	71.15 ± 4.02 ^a^	0.24 ± 0.01 ^c^	89.44 ± 9.42 ^c^	14.92 ± 2.23 ^bc^	68.08 ± 5.40 ^cd^	28.70 ± 14.37 ^a^
BC6	79.77 ± 13.63 ^a^	0.27 ± 0.05 ^c^	87.50 ± 14.57 ^c^	14.41 ± 2.21 ^c^	71.76 ± 9.33 ^c^	1.57 ± 0.23 ^b^
BC8	74.95 ± 1.07 ^a^	0.48 ± 0.01 ^b^	110.97 ± 3.64 ^b^	20.58 ± 1.20 ^a^	91.62 ± 3.60 ^b^	6.27 ± 5.25 ^b^
BBC	50.29 ± 2.12 ^b^	0.54 ± 0.05 ^b^	104.51 ± 3.45 ^b^	17.24 ± 0.36 ^b^	74.92 ± 3.03 ^c^	4.33 ± 1.12 ^b^

* P-Con: positive control, N-Con: negative control, BC6: treatment with viable cells (10^6^ CFU mL^−1^) of *B. subtilis* PE7, BC8: treatment with viable cells (10^8^ CFU mL^−1^) of *B. subtilis* PE7, BBC: treatment with broth culture of *B. subtilis* PE7. Data represent mean ± standard deviation of three replicates. Different letters in the same column indicate significant difference between treatments as compared by the least significant difference (LSD) test at *p* < 0.05 level.

**Table 3:** Effect of viable cells and broth culture of *B. subtilis* PE7 on micronutrient uptake in melon plants at 5 weeks after transplantation.

Treatment *	Micronutrient (mg plant^−1^)				
	Mn	B	Cu	Fe	Mo
P-Con	0.44 ± 0.07 ^a^	0.22 ± 0.15 ^a^	0.48 ± 0.12 ^a^	3.02 ± 1.15 ^c^	ND
N-Con	0.27 ± 0.05 ^b^	0.07 ± 0.03 ^b^	0.15 ± 0.06 ^b^	1.44 ± 0.38 ^ab^	ND
BC6	0.30 ± 0.08 ^b^	0.05 ± 0.02 ^b^	0.09 ± 0.05 ^b^	3.42 ± 1.31 ^a^	ND
BC8	0.40 ± 0.10 ^ab^	0.05 ± 0.01 ^b^	0.25 ± 0.17 ^b^	3.76 ± 1.34 ^a^	ND
BBC	0.37 ± 0.06 ^ab^	0.11 ± 0.03 ^ab^	0.23 ± 0.14 ^b^	2.64 ± 1.30 ^ab^	ND

* P-Con: positive control, N-Con: negative control, BC6: treatment with viable cells (10^6^ CFU mL^−1^) of *B. subtilis* PE7, BC8: treatment with viable cells (10^8^ CFU mL^−1^) of *B. subtilis* PE7, BBC: treatment with broth culture of *B. subtilis* PE7. ND means “Not detected”. Data represent mean ± standard deviation of three replicates. Different letters in the same column indicate significant difference between treatments as compared by the least significant difference (LSD) test at *p* < 0.05 level.

## Data Availability

All the data are available within the manuscript.
